# Supplementary material for: Polymorphisms in Glyoxalase I Gene Are Not Associated with Glyoxalase I Expression in Whole Blood or Markers of Methylglyoxal Stress: The CODAM Study
Source: Antioxidants (Basel). 2021 Feb 2;10(2):219. doi: 10.3390/antiox10020219 (PMC7913097; doi:10.3390/antiox10020219)
Supplement: Supplementary file 1 [file antioxidants-10-00219-s001.pdf]

Supplementary tables and figures

Supplementary Table 1 – General characteristics of the study population [1]

	Total population (n=546)
Age (years)	60 ± 7
Sex (%men)	61
NGM/IGM/T2DM (%)	52/23/25
BMI (kg/m2)	29 ± 4
Waist circumference (cm)	99 ± 12
Systolic blood pressure (mmHg)	140 ± 19
Diastolic blood pressure (mmHg)	82 ± 9
Fasting plasma glucose (mmol/L)	5.6 [5.2-6.4]
HbA1c (%)	6.0 ± 0.8
eGFR (ml/min/1.73cm³) [2]	91.1 ± 18.4
Triglycerides (mmol/L)	1.4 [1.0-2.0]
Total cholesterol (mmol/L)	5.2 ± 1.0
HDL cholesterol (mmol/L)	1.2 ± 0.3
Anti-hypertensive medication (%)	38
Lipid-mediating medication (%)	19
Glucose-lowering medication (%)	13
Total activity score (10^3 METs/week)	5.9 [3.7-8.7]
Current smokers (%)	23
Total alcohol intake (g/d)	8.5 [1.3-22.5]
Total energy intake (kcal/d)	2213 ± 666
Plasma D-lactate and MGO	
Fasting plasma D-lactate (µmol/L)	8.7 [6.3-12.9]
Fasting plasma MGO (nmol/L)	366.3 ± 77.8
iAUC MGO during OGTT	9413 [4042-14525]
Plasma AGEs	
Fasting plasma free CEL (nmol/L)	45.4 [36.6-58.4]
Fasting plasma free MG-H1 (nmol/L)	123.6 [86.6-172.6]
Fasting plasma protein-bound CEL (nmol/mmol lysine)	23.1 [18.7-28.9]
Urinary AGEs and D-lactate	
Urinary CEL (nmol/mmol creatinine)	517 [408-642]
Urinary MG-H1 (nmol/mmol creatinine)	2266 [1593-3300]
Urinary D-lactate (nmol/mmol creatinine)	0.8 [0.4-1.7]

[1]Data are presented as mean ± standard deviation for normally distributed variables, median (interquartile range) for skewed variables or proportion (%) for categorical variables.
[2]Calculated using the Modification of Diet in Renal Disease (MDRD) formula.
AGE, advanced glycation endproduct; CEL, Nε-(1-carboxyethyl)lysine; eGFR, estimated glomerular filtration rate; iAUC MGO, incremental area under the curve of MGO after an oral glucose tolerance test; IGM, impaired glucose metabolism; MG-H1, Nδ-(5-hydro-5-methyl-4-imidazolon-2-yl)-ornithine; MGO, methylglyoxal; NGM, normal glucose metabolism; OGTT, oral glucose tolerance test; T2DM, type 2 diabetes.

Supplementary table 2 – Means of Glo1 mRNA and markers of MGO stress for each of the three genotypes of the nine SNPs

SNP	Genotype	Glo1 expression		Plasma MGO and D-lactate					Plasma AGEs				Urinary AGEs and D-lactate				
		n	Glo1 mRNA	n	Plasma MGO	Plasma D-lactate	n	iAUC MGO post-OGTT (units)	n	Plasma free CEL	Plasma protein-bound CEL	Plasma free MG-H1	n	Urinary CEL	Urinary MG-H1	n	Urinary D-lactate
SNP4 rs3199033	AA	355	0.95 [0.74-1.2]	423	366 ± 78	8.4 [6.4-12]	376	9.8 [4.1-15]	423	45 [37-57]	23 [19-29]	123 [85-173]	396	512 [405-629]	2292 [1596-3202]	398	0.78 [0.38-1.6]
	AT	82	0.82 [0.58-1.0]	102	365 ± 72	9.5 [5.6-14]	93	8.1 [3.5-13]	102	46 [38-60]	24 [19-31]	118 [87-157]	97	532 [443-707]	2141 [1603-3331]	97	0.78 [0.44-2.1]
	TT	7	0.82 [0.59-1.0]	7	426 ± 72	9.1 [4.7-15]	6	6.4 [1.4-9.9]	7	60 [35-71]	21 [15-25]	158 [63-211]	7	547 [323-832]	2046 [1239-3557]	7	0.35 [0.27-1.8]
SNP13 rs3799703	GG	121	0.86 [0.60-1.1]	148	363 ± 73	7.9 [5.4-11]	140	8.4 [2.7-14]	148	46 [36-59]	23 [19-29]	122 [87-168]	141	484 [424-662]	2274 [1551-3330]	141	0.75 [0.38-1.7]
	AG	206	0.93 [0.71-1.2]	249	368 ± 82	9.0 [7.0-14]	216	9.4 [4.2-15]	249	45 [36-56]	23 [19-29]	120 [85-170]	237	520 [397-642]	2217 [1586-3459]	239	0.81 [0.39-1.8]
	AA	110	0.97 [0.76-1.2]	126	368 ± 74	7.9 [5.9-7.9]	111	11 [4.6-15]	126	45 [39-57]	27 [19-29]	123 [89-169]	115	522 [401-623]	2165 [1593-3032]	115	0.80 [0.38-1.5]
SNP16 rs2736655	GG	326	0.92 [0.68-1.1]	397	368 ± 80	8.7 [6.4-13]	354	8.9 [4.0-14]	397	45 [36-58]	23 [19-29]	121 [85-170]	374	511 [405-639]	2274 [1569-3368]	376	0.80 [0.38-1.7]
	AG	107	1.0 [0.73-1.2]	127	365 ± 72	8.9 [6.4-14]	113	10 [4.0-16]	127	46 [38-59]	24 [19-29]	129 [91-193]	120	532 [418-652]	2208 [1649-3264]	120	0.85 [0.40-1.7]
	AA	11	0.96 [0.78-1.3]	11	349 ± 51	7.3 [5.4-12]	10	8.5 [4.6-13]	11	41 [35-63]	27 [20-29]	135 [82-173]	9	429 [429-522]	2358 [1888-2605]	9	0.58 [0.41-2.5]
SNP18 rs2736654	TT	135	0.92 [0.74-1.2]	161	376 ± 73	9.3 [6.2-12]	143	9.9 [4.8-14]	161	46 [39-59]	24 [20-30]	121 [87-178]	149	525 [397-657]	2165 [1596-3246]	149	0.77 [0.43-1.4]
	GT	230	0.94 [0.68-1.2]	277	363 ± 80	8.5 [6.4-14]	242	8.8 [4.1-15]	277	45 [36-57]	22 [18-27]	126 [88-170]	262	516 [405-636]	2263 [1666-3315]	265	0.81 [0.36-2.1]
	GG	80	0.90 [0.70-1.2]	98	363 ± 73	8.3 [6.5-11]	93	9.5 [2.4-15]	98	47 [36-59]	24 [19-30]	115 [85-176]	92	492 [419-665]	2311 [1497-3477]	91	0.77 [0.40-1.6]
SNP28 rs3778443	GG	381	0.92 [0.71-1.2]	458	366 ± 70	8.5 [6.2-13]	412	9.5 [4.2-15]	458	46 [37-58]	23 [19-29]	124 [88-171]	428	518 [409-642]	2292 [1588-3281]	430	0.79 [0.38-1.7]
	AG	63	1.0 [0.68-1.3]	76	375 ± 82	9.7 [6.8-13]	63	8.6 [3.7-15]	76	48 [38-60]	23 [19-29]	126 [84-194]	73	519 [399-654]	2126 [16449-3489]	73	0.75 [0.36-1.4]
	AA	4	1.0 [0.71-1.4]	4	382 ± 30	9.5 [5.9-20]	4	6.7 [-1.6-16]	4	48 [35-69]	24 [20-28]	110 [87-218]	4	482 [350-540]	2479 [1244-3212]	4	0.54 [0.35-0.93]

SNP	Genotype	Glo1 expression		Plasma MGO and D-lactate					Plasma AGEs				Urinary AGEs and D-lactate				
		n	Glo1 mRNA	n	Plasma MGO	Plasma D-lactate	n	iAUC MGO post-OGTT (units)	n	Plasma free CEL	Plasma protein-bound CEL	Plasma free MG-H1	n	Urinary CEL	Urinary MG-H1	n	Urinary D-lactate
SNP38<br>rs1781716	GG	354	0.93<br>[0.71-1.2]	428	365 ± 77	8.4<br>[6.2-13]	386	9.1<br>[4.0-14]	428	45<br>[36-58]	23<br>[19-29]	121<br>[89-173]	402	517<br>[405-638]	2292<br>[1584-3259]	402	0.78<br>[0.38-1.7]
	CG	79	0.94<br>[0.68-1.3]	96	372 ± 81	9.6<br>[6.8-13]	81	10<br>[4.2-14]	96	49<br>[37-60]	23<br>[19-29]	132<br>[85-180]	90	528<br>[416-665]	2126<br>[1662-3637]	92	0.81<br>[0.47-1.5]
	CC	4	1.0<br>[0.71-1.4]	4	382 ± 30	9.5<br>[5.9-20]	4	6.7<br>[1.6-16]	4	48<br>[35-69]	24<br>[20-28]	110<br>[87-219]	4	482<br>[350-540]	2474<br>[1244-3123]	4	0.54<br>[0.35-0.93]
SNP40<br>rs10484854	CC	223	0.94<br>[0.73-1.2]	273	369 ± 75	9.0<br>[6.2-13]	242	9.5<br>[4.2-15]	273	46<br>[38-58]	24<br>[19-30]	120<br>[88-170]	252	525<br>[401-646]	2252<br>[1664-3263]	254	0.81<br>[0.44-1.7]
	CT	194	0.93<br>[0.66-1.2]	226	364 ± 79	8.5<br>[6.5-13]	201	9.3<br>[3.9-16]	226	44<br>[36-58]	23<br>[19-29]	126<br>[84-171]	216	511<br>[415-635]	2228<br>[1532-3142]	216	0.77<br>[0.35-1.7]
	TT	28	0.84<br>[0.73-1.1]	35	374 ± 83	8.3<br>[5.9-12]	33	7.9<br>[16-12]	35	50<br>[35-59]	24<br>[19-30]	143<br>[85-205]	33	441<br>[384-567]	2291<br>[1370-3505]	33	0.47<br>[0.28-1.2]
SNP45<br>rs17622621	GG	163	1.0<br>[0.78-1.2]	199	367 ± 74	8.8<br>[6.2-12]	174	10<br>[4.2-15]	199	45<br>[38-59]	24<br>[18-29]	120<br>[89-170]	181	525<br>[406-650]	2310<br>[1746-3213]	183	0.84<br>[0.44-1.6]
	AG	212	0.88<br>[0.67-1.2]	252	367 ± 82	8.5<br>[6.5-13]	226	9.5<br>[4.3-15]	252	44<br>[36-55]	23<br>[19-29]	118<br>[80-170]	241	511<br>[410-632]	2219<br>[1482-3265]	241	0.79<br>[0.38-1.7]
	AA	66	0.86<br>[0.70-1.1]	77	369 ± 71	8.7<br>[5.8-14]	72	7.3<br>[3.4-12]	77	51<br>[39-63]	24<br>[19-30]	130<br>[88-175]	73	511<br>[410-706]	2187<br>[1559-3377]	73	0.50<br>[0.33-1.7]
SNP49<br>rs1049346	AA	112	0.90<br>[0.68-1.1]	135	365 ± 74	8.8<br>[6.4-13]	122	8.3<br>[3.8-12]	135	50<br>[36-63]	23<br>[19-29]	126<br>[86-184]	127	511<br>[419-6845]	2125<br>[1667-3489]	128	0.62<br>[0.35-1.6]
	AG	240	0.94<br>[0.66-1.2]	284	370 ± 80	8.8<br>[6.5-13]	254	9.4<br>[4.0-15]	284	44<br>[37-55]	23<br>[19-29]	126<br>[84-170]	272	520<br>[408-637]	2327<br>[1500-3416]	272	0.79<br>[0.38-1.7]
	GG	94	0.97<br>[0.78-1.2]	117	364 ± 74	8.2<br>[6.0-13]	101	10<br>[4.1-16]	117	45<br>[37-59]	23<br>[18-28]	119<br>[89-170]	104	526<br>[406-663]	2319<br>[1909-3174]	105	0.91<br>[0.48-1.8]

[1]Data are presented as mean ± standard deviation for normally distributed variables and median [interquartile range] for skewed variables

AGE, advanced glycation endproduct; CEL, Nε-(1-carboxyethyl)lysine; iAUC MGO, incremental area under the curve of MGO after an oral glucose tolerance test; MG-H1, Nδ-(5-hydro-5-methyl-4-imidazolon-2-yl)-ornithine; MGO, methylglyoxal; OGTT, oral glucose tolerance test; SNP, single nucleotide polymorphism

Supplementary table 3 - Associations between iAUC MGO after an OGTT and SNPs of GLO1 using logistic regression

SNP	N	Genotype	iAUC MGO (low/high)	
			OR	p
SNP4 (rs3199033)	475	AA	-	-
		AT	0.82	0.40
		TT	0.39	0.32
SNP13 (rs3799703)	467	GG	-	-
		AG	1.00	0.99
		AA	1.38	0.24
SNP16 (rs2736655)	477	GG	-	
		AG	1.24	0.35
		AA	1.45	0.57
SNP18 (rs2736654)	478	TT	-	
		GT	0.82	0.36
		GG	0.89	0.68
SNP28 (rs3778443)	479	GG	-	-
		AG	1.01	0.99
		AA	0.30	0.33
SNP38 (rs1781716)	471	GG	-	-
		CG	1.23	0.40
		CC	0.32	0.36
SNP40 (rs10484854)	476	CC	-	-
		CT	0.90	0.60
		TT	0.98	0.96
SNP45 (rs17622621)	472	GG	-	-
		AG	0.91	0.66
		AA	0.72	0.26
SNP49 (rs1049346)	477	AA	-	-
		AG	1.30	0.26
		GG	1.48	0.17

Odds ratios of logistic regression, using a categorical variable of iAUC MGO (below/above median) as outcome variable and the three genotypes (dummy variables with major homozygous as reference) as main independent variable. Odds ratio indicates the odds of having a MGO concentration above the median for the stated genotype compared to the reference genotype. Results are displayed for fully adjusted model (adjusted for age, sex and glucose metabolism status). iAUC: incremental area under the curve. MGO: methylglyoxal. OR: Odds ratio.

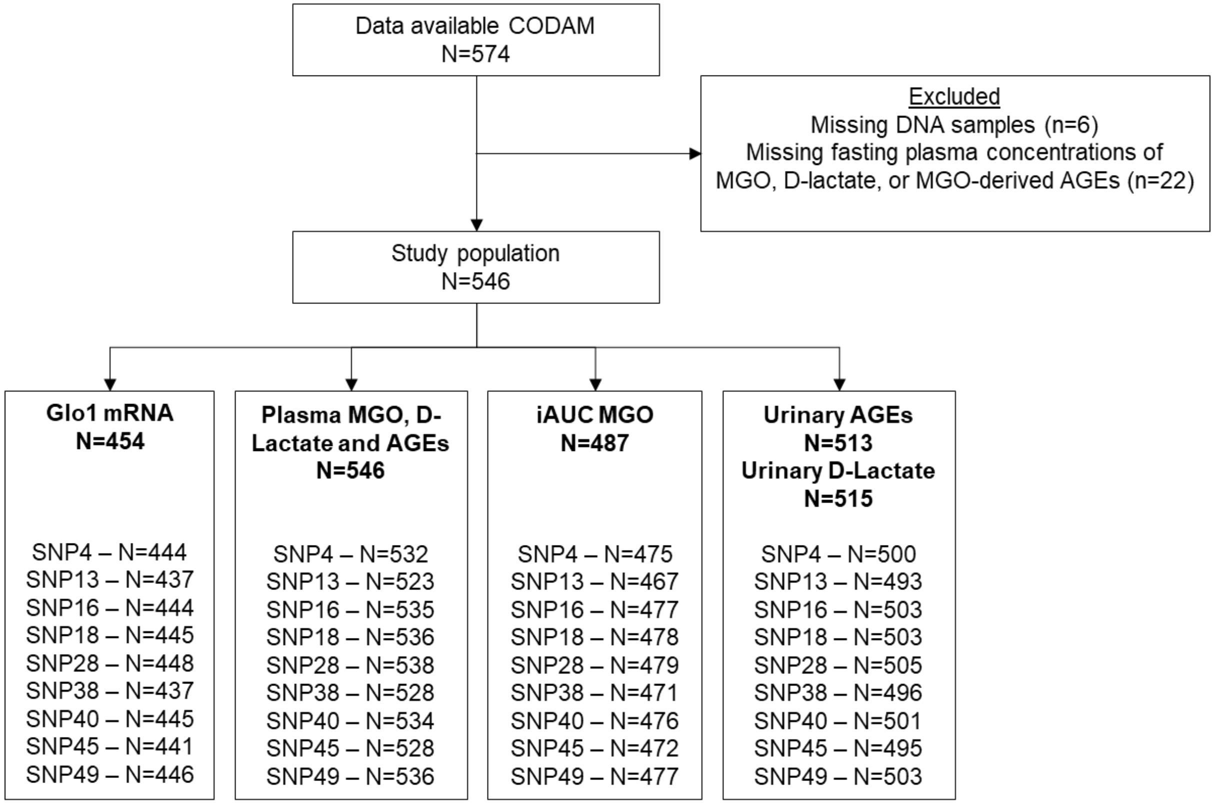

Supplementary Figure 1 – Flowchart of CODAM study population
